# A prospective cohort study on ambient air pollution and respiratory morbidities including childhood asthma in adolescents from the western Cape Province: study protocol

**DOI:** 10.1186/s12889-017-4726-5

**Published:** 2017-09-16

**Authors:** Toyib Olaniyan, Mohamed Jeebhay, Martin Röösli, Rajen Naidoo, Roslynn Baatjies, Nino Künzil, Ming Tsai, Mark Davey, Kees de Hoogh, Dilys Berman, Bhawoodien Parker, Joy Leaner, Mohamed Aqiel Dalvie

**Affiliations:** 1 Centre for Environmental and Occupational Health Research, School of Public Health and Family Medicine, University of Cape Town, Cape Town, South Africa; 2 Swiss Tropical and Public Health Institute, Socinstrasse 57, P.O. Box 4002, Basel, Switzerland; 3 University of Basel, Petersplatz 1, 4003 Basel, Switzerland; 4 University of Kwazulu-Natal, Durban, South Africa; 5 Cape Peninsula University of Technology, Cape Town, South Africa; 6 University of Washington, Seattle, WA 98195 USA; 7 Department of Environmental Affairs and Developmental Planning, Western Cape Government, Cape Town, South Africa

**Keywords:** Ambient air pollution, Environmental pollution, Outdoor air pollution, Traffic-related pollutants, Pollens, Childhood asthma, Respiratory morbidities, Allergic diseases, Lung function, Airway inflammation

## Abstract

**Background:**

There is evidence from existing literature that ambient air pollutant exposure in early childhood likely plays an important role in asthma exacerbation and other respiratory symptoms, with greater effect among asthmatic children. However, there is inconclusive evidence on the role of ambient air pollutant exposures in relation to increasing asthma prevalence as well as asthma induction in children. At the population level, little is known about the potential synergistic effects between pollen allergens and air pollutants since this type of association poses challenges in uncontrolled real life settings. In particular, data from sub-Sahara Africa is scarce and virtually absent among populations residing in informal residential settlements.

**Methods/design:**

A prospective cohort study of 600 school children residing in four informal settlement areas with varying potential ambient air pollutant exposure levels in the Western Cape in South Africa is carried-out. The study has two follow-up periods of at least six-months apart including an embedded panel study in summer and winter. The exposure assessment component models temporal and spatial variability of air quality in the four study areas over the study duration using land-use regression modelling (LUR). Additionally, daily pollen levels (mould spores, tree, grass and weed pollen) in the study areas are recorded. In the panel study asthma symptoms and serial peak flow measurements is recorded three times daily to determine short-term serial airway changes in relation to varying ambient air quality and pollen over 10-days during winter and summer. The health outcome component of the cohort study include; the presence of asthma using a standardised ISAAC questionnaire, spirometry, fractional exhaled nitric-oxide (FeNO) and the presence of atopy (Phadiatop).

**Discussion:**

This research applies state of the art exposure assessment approaches to characterize the effects of ambient air pollutants on childhood respiratory health, with a specific focus on asthma and markers of airway inflammation (FeNO) in South African informal settlement areas by considering also pollen counts and meteorological factors. The study will generate crucial data on air pollution and asthma in low income settings in sub-Sahara Africa that is lacking in the international literature.

## Background

Childhood asthma is the most common chronic disease in children globally and ranks in the top 20 contributors of global disability-adjusted life years (DALY) in all children; and among the top 10 causes of DALY amongst 5–14 year olds [1]. The global mortality rates of childhood asthma is approximately 0.7 per 100,000 [2]. Asthma impacts significantly on the quality of life of a child as it can interfere with physical exercise, results in school absenteeism as well as underperformance at school due to sleep interruption. It is estimated that two-thirds of asthmatic children suffer from a noticeable disability and approximately ten million school days are missed yearly [3]. Instances of severe asthma in children leading to frequent school absences might consequently affect the child’s education and probably career choice later in life. Furthermore, asthma in children creates a burden on the family since caregivers often take time ‘off-work’ to cater for sick children, hence a major cause of parental work absenteeism. Limitation of social life due to lack of sleep has been found in 50% of parents with asthmatic children and negligence of other siblings as more time is devoted to the asthmatic child [4].

Among other reasons, the increasing outdoor air pollution may also contribute to the increase in asthma prevalence in developing countries [5]. Ambient air pollution is a major environmental health concern globally affecting the population in both highly industrialised and developing countries. An estimated 23% of all deaths and 24% of the global burden of disease can be attributed to environmental factors, with ambient air pollution (especially particulate matter) estimated to be responsible for 3.2 million deaths per year (3.1% of global total DALYs) [5, 6]. In 2016, the WHO estimated that only about one in every ten people breathe clean air, according to the WHO Air Quality Guidelines [7]. The WHO further reported 3.7 million premature deaths in both urban and rural areas to be attributed to air pollution, which is mainly due to small particulate matter of 10 μm or less in diameter (PM_10_). Low-and middle-income countries (LMIC), where air pollution emissions from power-plants, traffic, open waste burning and other combustion sources are very common, account for about 88% of these deaths [7]. The WHO estimated that a 15% reduction in ambient air pollution-related deaths can be expected from reducing ambient particulate matter from 70 to 20 μg/m^3^ [7]. Respiratory diseases are the leading cause of death from ambient air pollution, responsible for over half of the deaths reported [7]. Of special concern is the effect of ambient air pollution on children as their immune systems and lungs are not fully developed with the onset of exposures in early-life [8, 9]. Furthermore, children tend to spend more time outdoors in parks and play grounds, and likely to breathe a greater amount of air pollutant per body weight compared to adults similarly exposed. Recent studies have suggested that the development of asthma, atopy and infant mortality is associated with air pollution, particularly traffic-related pollution [10]. The evidence for the extent to which air pollution affects children’s respiratory health is inconclusive suggesting much need for further investigation.

Various studies have demonstrated that exposure to ambient air pollutants in early childhood plays an important role in the exacerbation of asthma and other respiratory symptoms with greater effect amongst asthmatic children [11–16]. Furthermore, studies have demonstrated the association between ambient air pollution and lung function deficits and more recently on increased FeNO in children [17–24]. It has been postulated that continuous exposure to air pollutants, particularly outdoors where children spend most of their time, may exacerbate the impact of early childhood exposures that lead to asthma and other respiratory symptoms expression. However, it should be noted that the bulk of these evidence comes from regions outside sub-Sahara Africa. Furthermore, there is inconclusive evidence on the role of ambient air pollutant exposures in relation with increasing asthma prevalence as well as asthma induction in children [25, 26].

Although environmental and lifestyle factors appear to drive the increasing susceptibility to developing allergic diseases such as asthma, the increased susceptibility in response to pollen allergen in population exposed to high levels of pollution remain elusive. While the pathogenesis of asthma and allergies have demonstrated a link between combined exposure to air pollutants and pollens in in-vitro and animal studies, little is known about their synergistic effect at the population level [27]. A growing body of evidence has demonstrated a link between enhanced risk of atopic sensitization and exacerbation of symptoms due to the interaction between air pollutants and airborne allergens [27–29]. Nevertheless, more research is needed to clarify the mechanism by which air pollutants and pollens induce airway changes in exposed individuals. Some recent evidence have demonstrated that pollen allergen can also induce non-atopic reactions such as irritation and inflammation in addition to allergic responses such as immunoglobulin production [27]. However, very few epidemiological studies have taken into account separating allergic and non-allergic asthma and underlying mechanisms due to the common inflammatory component present in both mechanisms. To better evaluate the relative contribution of the role air pollution in allergic and non-allergic asthma, more research is needed since air pollution is considered to be a major risk factor for non-allergic respiratory diseases.

Most studies of the association between air pollution and childhood asthma have been performed in industrialised settings. Given the multifactorial nature of these diseases, the need for research is particularly eminent in LMIC where patterns of co-exposure, co-morbidities and susceptibility may largely differ from those in Western countries. The WHO also recommended in the 2016 report on the effect of ambient air pollution [7] that more epidemiological studies are needed to estimate the long-term health effect of air pollution especially in LMIC plagued with unacceptable levels of air pollution. Further studies in developing countries are needed so as to render comparison between South African and other developing countries to findings reported from other parts of the world, thereby contributing to the evidence on the associations between air pollution and asthma in children.

## Methods/design

### Aim & objectives

The aim of this prospective cohort study is to investigate the association between ambient air pollution and co-exposure to pollen with respiratory morbidities focusing on asthma among children in the Western Cape, South Africa.

The objectives are:

1. To determine the prevalence of asthma (defined by asthma symptom score, presence of airway obstruction on spirometry, and elevated FeNO) in primary school children residing mainly in informal settlement areas of the Western Cape with diverse exposure to air pollutants and airborne pollen.
2. To characterize baseline exposures, temporal trends and spatial distribution of ambient air pollutants and relevant airborne pollen and their association with meteorological parameters on their dispersion.
3. To examine the effect of air pollutants and pollen levels on asthma outcomes controlling for potential confounders and exploring the potential for effect modification:

- To examine the effect of daily variations in ambient air pollutants and airborne pollen levels across seasons on asthma outcomes (defined by respiratory symptoms and changes in serial peak flow and FEV_1_ measurements) in a panel study of participating children in the Western Cape.
- To examine the exposure-outcome relationships (at baseline and at the 12 month follow-up assessment) between exposure to ambient air pollutants and airborne pollen with asthma outcomes (defined by asthma symptom score, presence of airway obstruction on spirometry, and elevated FeNO).
- To explore longitudinal 12 month change in ambient air pollutants and airborne pollen on changes in asthma symptom score, lung function (FEV_1_ and FEV_1_/FVC ratio), and FeNO in this cohort.

4. To examine whether increased baseline FeNO predicts new onset asthma (defined by increasing asthma symptom score or presence of new asthma symptoms).

### Study population and design

The study areas includes three areas identified in a needs analysis conducted by the Western Cape Department of Environmental Affairs and Development Planning (DEA&DP) in 2013. The prioritised areas include an urban industrialised area in Cape Town (Milnerton/Milnerton Ridge including Marconi-Beam, Phoenix and Joe Slovo), a peri-urban area outside Cape Town with a large informal sector (Khayelitsha) and a rural area (Oudtshoorn) more than 400 km outside Cape Town. Additionally, an area (Masiphumulele, Noordhoek) with negligible ambient air pollutants exposure and community of similar socio-economic status as the prioritised areas is identified. These four study areas are selected to maximize contrasts in exposure levels to the different ambient air pollutants.

The study comprises of a baseline study and a 12-months follow-up study of the cohort in which all examinations including a child and caregiver questionnaire, lung function testing in the form of spirometry, exhaled nitric-oxide and allergy testing will be conducted. Additionally, a panel study is embedded investigating seasonal relationships between short-term variation in ambient air pollution and lung function measured through daily peak flow measurements in children during a 2-week period in summer and winter. This ensures a design whereby the exposures, potential confounding factors and health outcomes are monitored seasonally and repeatedly in a prospective manner.

### Sampling

In total 600 participants are selected from children attending primary schools located in the three prioritised and one less-exposed areas. A list of all schools in the study area is obtained from the Department of Education in the Western Cape Province. One or two schools from each area located nearest to the City of Cape Town metro air quality monitoring stations are selected. One hundred and fifty Grade-4 students in each study area are targeted. Grade-4 students are selected for the study since they will not leave primary school to enter senior school during the study period. The average age of Grade-4 students is 10 years; as such they are old enough to adhere to instructions during the various tests and reasonably confident to answer simple questions regarding their symptoms. This age group also provides a key phase in the onset of childhood asthma which often is preceded by atopic dermatitis and allergic rhinitis (with reference to the ‘atopic march’). Although, there are gender differences in asthma reported in the literature, all eligible Grade-4 students are included in the study and gender will be adjusted-for, stratified and assessed for interaction in the analyses.

After meeting the school principal, obtaining permission from the schools board, and obtaining the Grade-4 class lists and addresses, the houses of the school children are visited by trained field staff to obtain the caregivers (parent or guardian) consent. Random sampling is used to select 75 students from the list of consented children when the number of consented children exceeds 75 in each school.

All Grade-4 students attending selected schools qualify for inclusion in the study. Baseline data is collected between February 2015 and August 2015, while the follow-up data is obtained from May 2016 to September 2016. However, spirometry is rescheduled for participants with a positive response obtained from a pre-test questionnaire to any of the following items: If the child had a recent operation (in last 12 months); If the child feels like vomiting or has any pain; If the child is being treated for Tuberculosis; If the child had flu, sinusitis or lung infection in the last 3 weeks. However, children with positive response to ‘having epilepsy’ are excluded from doing spirometry.

### Questionnaire

Trained interviewers administered questionnaires to child participants and their caregivers in their spoken language in the cohort study. The use of mobile technology is implemented in the administration and capture of questionnaires. For interviews of child participants, the questionnaire contains items on demographic factors and respiratory health incorporating the standardized and validated International Study of Asthma and Allergies in Childhood (ISAAC) questionnaire [30]. For interview of caregiver, the questionnaire contains items on:

i) Demographic factors (age, gender, education) of child
ii) Birth history (Mother’s age at child’s birth, birthweight, maternal smoking during pregnancy)
iii) Diet (child’s nutritional habit)
iv) Health impairment (presence of other illness and vaccination profile)
v) Respiratory conditions and allergy (presence of respiratory symptoms such as cough, wheeze, shortness of breath and chest tightness, including other allergic conditions such as eczema, itch/rash, food allergy, pet allergy)
vi) School attendance (school absenteeism to assess the impact of respiratory illnesses such as asthma)
vii) Asthma severity (doctor-diagnosed asthma and other allergic diseases, presence of current wheeze)
viii) Health services utilization (regular hospital visits)
ix) Asthma medication
x) Exposure to indoor pollution and allergens (such as heating and cooking fuels, dust mites, cockroaches, rats, molds and pets)
xi) Household characteristics and hazards
xii) Respiratory health of the child based on the ISAAC questionnaire (assessing the presence of rhinoconjuctivitis) [30]
xiii) Child residential history to assess the duration of exposure in the sampling study area

The questionnaires are translated and back-translated. The questionnaires is administered at all phases of the study.

### Pulmonary function assessment

Pulmonary function assessments for the cohort study are conducted during school visits, comprising spirometry conducted according to the American Thoracic Society (ATS) guidelines [31] and fractional-exhaled nitric oxide measurement (FeNO). Spirometry and FeNO is conducted at baseline and in the follow-up period. Additionally, two intensive phases (once during peak winter and another during peak summer) of serial peak flow measurements are conducted bihourly per day during the school time over a two week period for all school child participants (panel study). During this period, school children are asked to complete a bihourly symptom activity log recording symptoms, odour, location and activity. The serial peak-flow measurement is conducted simultaneously at all schools.

#### Spirometry

An experienced technologist conducts spirometry using a flow-volume spirometer (EasyOne 2001–3 Spirometer). Spirometers are calibrated at least twice a day using a three-litre syringe. The temperature and humidity are monitored on a daily basis. The technologist is blinded to the exposure status of participants. Spirometry is performed in a sitting position with nose clips. Each child participant performs a maximum of up to eight trials to produce three acceptable curves. Test reproducibility is used as a guide to whether further attempts will be necessary. Reproducibility criteria is based on the two best tracings for both FEV_1_ and FVC varying by no more than 150 ml or 5%, whichever is greater. However, failure to meet reproducibility criteria does not result in exclusion of the spirogram results from the statistical analysis. Poor reproducibility may also be an independent marker of airway dysfunction [32]. The lung function indices of primary interest include forced vital capacity (FVC) and forced expiratory volume in one-second (FEV_1_). The best FEV_1_ and FVC is used regardless of whether they belong to the same tracing. Lung volumes obtained by spirometry are adjusted for body temperature and pressure according to the temperature and atmospheric pressure measured on a continuous basis throughout the day. Special instructions are given to participants to ensure that tested individuals do not take any anti-asthmatic inhalers (12 h before) or oral asthma medications (48 h before) prior to the test. Participants are administered an inhaled bronchodilator and have spirometry repeated after a minimum of 10-min waiting period. Special precautionary measures include having readily available oxygen and B_2_-adrenergic agents for nebulization. Additionally, emergency medical personnel are on-site or within quick access at all times during the tests. All participating children are assessed during school hours.

#### Fractional exhaled nitric oxide (FeNO)

Fractional-exhaled nitric oxide (FeNO) measurement is a recognized non-invasive method for assessing allergic airway inflammation [33]. The Fractional-exhaled nitric oxide (FeNO) testing is conducted by a trained nurse. A hand-held portable nitric oxide sampling device (NIOX MINO® Airway Inflammation Monitor; Aerocrine AB, Solna, Sweden) is used. The child is advised to sit comfortably and breathe normally for about five minutes to acclimatize, and thereafter instructed to inhale close to total lung capacity (TLC). This is followed by an immediate exhalation at a constant flow rate of 50 ml/s for at least four seconds. Two technically adequate measurements are performed (at least 30-s interval) in line with the American Thoracic Society/European Respiratory Society recommendations [34]. The average value of the two measurements is used in the analysis. Special instructions are provided to participants to ensure that tested individual avoids strenuous exercise, eat nor drink (at least an hour) prior to the test. The passive smoking history and date of last medication of bronchodilator is recorded. It is important to note that spirometric manoeuvres may reduce exhaled NO levels; hence FeNO measurements are done before spirometry. Furthermore, children with upper and lower respiratory tract infection have their measurements deferred until recovery or had their measurement taken with infection state recorded. The participants’ height and weight is measured and this information is used to compute the body mass index (BMI). Ambient NO and temperature are also recorded.

#### Panel study: Collection of bihourly symptoms log and peak flow data

In the panel study, each child is asked to perform serial peak flow measurements bihourly (just before school starts, during the 1st break and 2 h thereafter) and complete an activity symptoms log in between serial peak flow measurements on each school day over each of the 2-week sampling phases (summer and winter phases). The activities recorded in the symptoms log include symptoms, odour detection and information about location during the previous 2 h. Three consecutive manoeuvres of peak expiratory flows are performed by each child immediately prior to completion of the bihourly logs using an individual Microlife Peak flow meter (Microlife PF100). Each child is trained to use the device before fieldwork starts and reminded daily during both phases by trained research assistants. The Microlife PF100 meets the ATS standards, and it is easy to use on children due to its user-friendly interface. The device measures both Peak Expiratory Flow (PEF) and FEV_1_. This procedure is largely dependent on the child’s cooperation, efforts and ability to inhale maximally. To obtain a maximum value for the PEF with a rapid upstroke, the children are encouraged to blow vigorously as they physically can. To avoid measurement errors, the children are advised to blow out quickly, and avoid flexing the neck, as this might lead to the PEF dropping by 10% due to visco-elastic properties of the trachea [35]. Testing are conducted simultaneously at all schools according to the American Thoracic Society/European Respiratory Society (ATS/ERS) guidelines. Trained research assistants are present at each school at all times to oversee the measurements.

### Assessment of allergic status

#### Allergy tests: Phadiatop levels

Samples of blood (9 ml) are drawn from each participant using a Becton Dickinson Vacutainer SST tube (with gel medium and clot activator) by the nurse. Each sample is labelled and stored in a cooler box at 4-degrees Celsius before being transported to the laboratory for spinning down. The blood is allowed to clot for 1–2 h at room temperature (20–24 degrees Celsius) and then centrifuged at 1350 rpm for 10-min at room temperature. The serum is then transferred to another tube and stored at −20-degrees Celsius in a freezer. These serum samples are then transferred to the main freezers where they are stored at −80-degrees Celsius. The samples are then couriered to the SANAS accredited (ISO 15189:2007), National Institute for Occupational Health (NIOH) Immunology laboratory for immunological analysis. Presence of sensitization to common aeroallergens (house dust mites, grass pollens, cat, dog, and cockroach) are determined by the Phadiatop® test (Phadia AB, Uppsala, Sweden). The analysis is done blinded to further details of the participant. The Phadiatop test is conducted in the baseline study as well as in the follow-up period. A positive result would be indicative of the presence of atopy.

### Exposure characterisation

#### Ambient air pollutants

Exposure to ambient air pollution is estimated from a) geographic information system-based indicators b) air monitoring data from the stationary monitors in each of the study areas, c) available dispersion and chemical modelling, d) fine-scale land-use regression modelling based on two air pollution measurement campaigns in winter and summer in each study area at a total of 130 homes of study participants.

- ***Geographic information system (GIS)-based measures***: GIS-based indicators are extracted as an exposure proxy for point and mobile sources of air pollution such as traffic-related pollutant (NO_2_ and PM_2.5_). The distance of each participants’ address to point sources, local and major roads, highways is computed using ArcGIS software. In addition, several buffers (50 m, 150 m and 200 m) are created around each participant’s address, and the total roadway length is calculated using ArcGIS. It should be noted that roadway length within each buffer can also be weighted-by the average traffic frequency and vehicular types plying each roadway (depending on data availability). Additionally, other GIS data may also be used such as land use/classification, street network, building and population density which would also be calculated for different buffer sizes.
- ***Fixed-site air monitoring data***: Ambient air sulphur dioxide (SO_2_), nitrogen dioxide (NO_2_), Particulate Matter of 2.5 μm in diameter (PM_2.5_), Particulate matter of 10 μm in diameter (PM_10_), and ozone (O_3_) measurements from the City of Cape Town’s and DEA&DP stationary air quality monitoring stations in each study area are obtained throughout the study period. The sampled air quality data are stored in the City of Cape Town’s Data Acquisition System (DAS). The storing, pre-validation and transmission of data on the Air Quality Monitoring Network is the responsibility of the Western Cape Department of Environmental Affairs and Development Planning. Most pollutants are measured at seconds-to-minutes resolution using the US Environmental Protection Agency (EPA) approved methods and the values are expressed as one-hour averages.
- ***Dispersion and chemical modelling***: Dispersion and chemical modelling is used to estimate air pollution levels over specific time periods across the different study areas. Input data for these models are emissions from major sources such as industry, household or domestic fuel-use obtained from household surveys, traffic estimated from the number and types of vehicles in the areas, biomass fuel burning and meteorological variables (such as wind speed, wind direction, temperature, humidity, and precipitation). High resolution distribution of primary pollutants concentrations is provided by the dispersion modelling, while concentrations of secondary pollutants such as ozone and particulates are derived from the chemical modelling. CALPUFF system is used to create the dispersion modelling at a resolution of 300 m over the study areas [36]. This system captures complex terrain and diurnal wind regimes such as land-sea breezes more effectively. The model is based on the principle that meteorological factors influences the direction and dispersion of pollutants from their point sources, and thus residential exposure depends on their location upwind versus downwind of the point sources. On the other hand, chemical modelling is used to model pollutant transformation in addition to secondary formation of aerosols which may impact on the concentrations of PM_2.5_ in the Province. A comprehensive Air Quality Model with Extensions (CAMx) is used to model chemical pollutants due to its suitability for the integrated assessment of particulate and gaseous air pollution over many temporal scales. The data from the model is used to estimate the average exposures at each participant’s residence in the short-, medium-, and long-term through the study period (daily, weekly, monthly and annually). These model output are however highly dependent on the quality of their input emission data.
- ***Land-use regression (LUR) modelling***: To refine the spatial resolution of exposures at the individual level, Land Use Regression (LUR) models are used to estimate the spatial distribution of air pollutant concentrations across each study area and for all relevant locations. This is based on ambient air pollutant measurements in all four study areas using passive diffusion samplers to measure NO_2_, SO_2_ and O_3_ and active pump-based 37 mm filter samples (PM_2.5_, PM_2.5_ absorbance). In addition, real-time continuous air pollutant measurements (NO_2_, O_3_, CO, PM_10_, PM_2.5_, and PM_1.0_) is recorded at the schools and reference site per study area. In three study areas, 40 houses per study area including participating schools and a background reference site are selected to represent the full range of exposure levels within the study area. However, for the Noordhoek study area, a total of 20 houses is selected due to its small geographical size. Measurements in each selected house are conducted for a week, over a 4-week sampling period per area, while measurements at schools and the reference site are done weekly throughout the entire 4-week sampling period. GIS-based model predictors are derived from ArcGIS software described above. Temporal adjustment within each and across both seasonal measurement campaigns (summer and winter) are done by using data from schools, reference sites and from the City of Cape Town’s and DEA&DP air quality monitoring stations including meteorological data from the South African Weather Service (SAWS). Additionally, questionnaire responses on ambient and indoor air pollution (such as heating and cooking fuels), including dust mites, pets, moulds and use of pesticides at home are considered in the exposure assessment.

### Pollen measurements

Tree pollen, grass pollen, weed pollen and mould spores are measured in all the four study areas from July 2015 to March 2017, using the Burkard 7-day recording volumetric Spore Trap which allows for continuous spore trapping. There are varying concentrations of pollen and spore at heights above ground level, thus the trap is placed above the ground level (ideally a flat rooftop) at a minimum height of 1.5 m to avoid dust contamination. Pollen and fungal spores are measured at each study area for one-calendar year in order to properly assess the seasonal peaks of the local aeroallergens. The average daily concentration is expressed as particles/m^3^ using the concentration conversion below;

$$ \frac{\mathrm{N}\times \mathrm{total}\  \mathrm{area}\  \mathrm{of}\  \mathrm{strip}\ \left(48\mathrm{mm}\times 14\mathrm{mm}\right)}{\mathrm{Area}\  \mathrm{of}\  \mathrm{stip}\  \mathrm{analysed}\ \left(\mathrm{field}\  \mathrm{diameter}\times \mathrm{length}\  \mathrm{of}\  \mathrm{traverse}\ \mathrm{x}\ \mathrm{no}\ \mathrm{of}\  \mathrm{traverse}\mathrm{s}\ \mathrm{x}\ 14.4\right)} $$

Where N = the pollen or fungal spore concentration per m^−**3**^ **.**

14.4 = volume of air sampled per 24 h period, at 10^**−1**^ l/min.

Field diameter = width of microscope field.

### Meteorological factors

Meteorological factors such as rainfall, sunshine, temperature, barometric pressure, humidity, wind speed and wind direction are obtained (minimum, maximum and daily averages) from the South African Weather Service (SAWS). These factors are considered in the LUR models as well as in the statistical analyses of the associations between ambient air pollutants and respiratory health outcomes in children. These factors have been found to influence the spatial and temporal distribution, deposition and formation (in the case of secondary pollutant such as ozone), of pollutants including the altered spatial and temporal distribution of allergens (pollens, moulds and house dust mite) [27, 37]. Meteorological factors have also been shown to influence the exacerbation of allergic respiratory conditions including asthma especially in vulnerable groups such as children [27].

### Statistical analysis

#### Sample size calculation

The sample size required for the study was calculated using the following formula below;

$$ \frac{\mathrm{N}\approx 4\mathrm{P}\ \left(1\hbox{-} \mathrm{P}\right)\ \left({\mathrm{Z}}_{\alpha /2}+{\mathrm{Z}}_{\beta}\right)}{{\left(\mathrm{RD}\right)}^2} $$

Where N = sample size, Z_α/2_ is the alpha risk, and Z_β_ is the power

$$ \frac{\mathrm{P}=\kern0.5em {\mathrm{P}}_0+{\mathrm{P}}_1}{2} $$

P_0_, the prevalence of asthma is urban areas of Cape Town estimated at 34.4% [38].

P_1_, the prevalence of asthma amongst exposed school children, 48.8% found in a study conducted in Durban, South Africa [39].

Therefore $$ \frac{\mathrm{P}=0.344+0.488=0.416}{2} $$.

RD is the risk difference P_1_ – P_0_ = 0.488–0.344 = 0.144.

Using an alpha risk of 0.05 and a power of 80% (β = 0.20).

Thus, $$ \frac{\mathrm{N}\approx 4\ (0.416)\ \left(1\hbox{-} 0.416\right)\ \left(1.96+0.842\right)}{(0.144)^2}=368 $$

The approximate sample size calculated for the study is therefore 368.

### Outcome variables

#### Doctor-diagnosed asthma

A positive response by the parent or guardian to the question in the questionnaire, ‘if the child participant was previously diagnosed by a doctor to have asthma’ and treated as a dichotomous variable (Yes, No).

#### Asthma symptoms scores

The operational definition of asthma based on asthma symptoms will be a sum of positive answers to eight main asthma symptoms and bronchial hyper-responsiveness questions from the Child Caregiver question, which generates a continuous asthma score ranging from 0 to 8 [40]. Continuous asthma scores will be produced from the following questions below;

1. How many episodes of wheezing or whistling has the child had in the past 12 months?
2. In the past 12 months, how many times has the child had wheezing that made it hard for him or her to breathe or catch his or her breath?
3. In the past 12 months, how many times has the child complained that his or her chest felt tight or heavy?
4. Does the child get short of breath walking with other children of his/her own age on level ground?
5. In the past 12 months, how many times has the child coughed with exercise or running or playing hard?
6. In the past 12 months, how many times has the child’s sleep been disturbed due to wheezing, coughing, chest tightness or shortness of breath?
7. Has a doctor or nurse ever told you that the child has asthma?
8. Has the child ever taken any medication for asthma?

#### Airway obstruction

Airway obstruction will be defined as a reduced FEV_1_ (less than 80% of the predicted value- using the multi-ethnic reference values for spirometry for the 3–95 year age range [41]) and a reduced FEV_1_/FVC ratio (less than 0.8) recorded as a dichotomous variable (Yes, No).

#### Airway inflammation

Airway inflammation will be assessed using the fractional exhaled nitric oxide (FeNO) levels, which is a marker of eosinophilic airway inflammation. Elevated levels of FeNO have been implicated in the detection of subclinical airway inflammation, even in the absence of lung function impairment and absence of asthma symptoms [42]. Children with FeNO levels less than 15 ppb will be considered normal; those with levels between 15 and 35 ppb will be categorized as ‘elevated’, while children with FeNO levels above 35 ppb will be categorized as having ‘high’ levels [42].

#### Atopy

Atopy will be defined as individuals with serum Immunoglobulins E (IgE) antibodies above 0.35kUA/l on the Phadiatop test. Although this measurement does not provide information on the specific allergens the individual is sensitized-to, it does give an indication of the presence of an allergic sensitization to common inhalant allergens. Serum IgE levels are also linked to airway hyper-responsiveness in the presence or absence of asthma symptoms [38].

#### Variability in PEF and FEV_1_

Increased intraday variability and lower nadir values (minimum best or daily lowest valid values) are markers of worsening of asthma. Thus, the average within-day variability in PEF and FEV_1_ over the 10-days in each sampling season (summer and winter) will be calculated for each participant including the average minimum best values of PEF and FEV_1_ over the 10-days.

#### Rapid decliners in FEV_1_

This will be used as another marker of airway obstruction and defined as children whose FEV_1_ reduces by 30 ml/year (the upper limit of physiological decline) and/or children with deficit in lung function growth. The deviation from the normal lung function growth curve will also be explored using the multi-ethnic reference values for spirometry for the 3–95 year age range [41].

#### New-onset asthma

A new asthma case will be defined as a child with no prior parental report of a physician diagnosis of asthma in the baseline survey whose parent or guardian reports a doctor diagnosis of asthma in the follow-up year. Additionally, participants defined as rapid decliners in FEV_1_ on follow-up and/or participants with appreciable changes in the asthma symptom score will also be classified as new asthma cases.

### Statistical analysis protocol

#### Analysis protocol for objectives 1

Descriptive analyses is used to examine the characteristics of the study population, to characterize the asthma symptom score (ASS), the distribution of the various pulmonary function tests (PFT), and FeNO measurements during year-1. The ASS is based on the sum of positive answers to eight main asthma symptoms and bronchial hyper-responsiveness questions, while airway obstruction and airway inflammation is based on definitions previously described. Univariate linear, logistic, Poisson or ordered logistic regression analyses will be used to examine the association between variables, including demographic characteristics, personal characteristics, and household characteristics with respiratory outcomes including allergic status, ASS, spirometric indices and FeNO.

#### Analysis protocol for objectives 2

The mean annual levels and seasonal variations, annual and seasonal exceedances of South African Ambient Air Quality Standards and annual maximum levels in NO_2_, SO_2_, PM, and O_3_ in each area will be determined from continuous data from ambient air quality monitoring stations during the study period and also dispersion modelling data. Annual and seasonal averages of NO_2_, SO_2_ and PM, will be determined from average 24-h levels, while for O_3_, an eight-hour daytime average (from 10 a.m. to 6 p.m.) and of the one-hour maximal levels will be computed. Missing data (e.g. from technical failures) in ambient air quality monitoring data will be imputed based on regression models using measurement data from other close monitoring sites and meteorological variables. Furthermore, spatial variation in pollutants in each area will be determined using LUR as described above. The LUR models constructed for the time period of the measurement campaigns will be extrapolated to other time windows including long-term averages by using data measurements from the stationary air quality monitoring stations within each area as well as meteorological data.

The total airborne pollen measured in all four study areas will be described as a continuous variable and later categorize as follows; Tree pollen in count/m^3^ ranging from 0 to 15 ‘low’, 16–90 ‘moderate’, 91–1500 ‘high’ and above 1500 ‘very high’; Grass pollen in count/m^3^ ranging from 0 to 5 ‘low’, 6–20 ‘moderate’, 21–200 ‘high’ and above 200 ‘very high’; Weed pollen in count/m^3^ ranging from 0 to 10 ‘low’, 10–50 ‘moderate’, 51–500 ‘high’ and above 500 ‘very high’; and mould spores in count/m^3^ ranging from 0 to 900 ‘low’, 900–2500 ‘moderate’, 2500–25,000 ‘high’ and above 25,000 ‘very high’. Time-series analysis will be used to investigate the seasonality of each airborne pollen count while their correlation between meteorological variables such as temperature, relative humidity, rainfall and wind speed and direction two to 3 days before measured levels will also be explored.

### Analysis protocol for objectives 3

i) ***Protocol for objectives 3a.***

*Exposures*: In the panel study, the hourly and daily averages of each pollutants (24-h averages for PM, SO_2_ and NO_2_, and 8-h average for O_3_) and airborne pollens will be determined for the same day as well as for a lag period of 7-days.

*Outcomes*: Daily nadir (minimum best) and intraday variability of peak expiratory flow (PEF) and FEV_1_ will be used as markers for the worsening of asthma.

*Covariates*: The potential confounders determined *apriori* include: age, gender, race/ethnicity, asthma medication use, respiratory allergy history, atopic status, BMI, hour and day (of the week) of PEF, season, and temperature. Other covariates with *P-value < 0.20* from the bivariate analysis will also be included in the multivariate model.

*Analytical method*: A general additive multiple linear regression model (GAM) will be fitted for predicting the percent change in within day PEF and FEV_1_, and nadir PEF and FEV_1_ for an interquartile range (IQR) increase in particular pollutant and airborne pollen while taking into consideration possible confounders and effect modifiers including penalized splines for seasonal and meteorological variations. The IQR scaling will enable the percentages to be directly relevant to the exposure experienced by the participants and will also make the percentages for different pollutants directly comparable to each other. To account for within-subject correlation, Generalized Estimating Equation (GEE) regression model will also be used as it focuses on estimating the average response through the fixed-effect or random-effect, as opposed to a regression parameters that predicts the effect of changing one or more covariates on a given participant.

ii) ***Protocol for objectives 3b.***

*Exposures*: The hourly and daily averages of each pollutants (24-h averages for PM, SO_2_ and NO_2_, and 8-h average for O_3_) and airborne pollens will be used to construct a lag of up to 30 days. The annual averages of air pollutants from the LUR will also be considered.

*Outcomes*: Asthma symptom score (ASS) will be on an ordinal scale ranging from 0 to 8 as defined above. Airway obstruction will be defined as a dichotomous variable with reduced FEV_1_ (less than 80% of the predicted value) and a reduced FEV_1_/FVC ratio (less than 0.8). Airway inflammation will be assessed using the log-transformed FeNO levels on a continuous scale and also as a polychotomous (ordered) variable as categorized above.

*Covariates*: The potential confounders include; age, gender, race/ethnicity, asthma medication use, respiratory allergy history, atopic status, BMI, and day (of the week) of test date, season, and temperature. Other covariates with *P-value < 0.20* from the bivariate analysis will also be included in the multivariate model.

*Analytical method*: Multiple linear regression will be used to model the associations between IQR range increase (or a 10 μg/m^3^ increase where applicable) in pollutants (air pollutants and airborne pollen) and continuous outcome of interest while taking into consideration possible confounders and effect modifiers. Multiple logistic regression will be used in the case of a binary outcome while multiple ordinal regression will be used for ordinal outcomes such as ASS. In addition, to examine the co-dependency of the various pollutants, the correlation structure will be used to guide the selection of a two-pollutant model to avoid potential multi-collinearity. Sensitivity analysis on the lag structure will also be done using different exposure windows up to 60 days prior to examinations, whenever appropriate, to evaluate the temporality of possible effects. For the purpose of this analysis, long-term effects of pollution levels will be investigated as potential confounders or effect modifiers of the short-term effects in models that will include random intercepts.

iii) ***Protocol for objectives 3c.***

*Exposure*: The change in annual averages of 24-h period for NO_2_, SO_2_ and PM, and 8-h daytime average for O_3_, including airborne pollen levels across the cohort study period.

*Outcomes*: Changes in ASS at follow-up, changes in levels of FeNO (∆FeNO) or percentage predicted FEV_1_ (∆%FEV_1_) and FEV_1_/FVC ratio (∆FEV_1_/FVC) between the two study periods.

*Covariates*: The potential confounders include; age, gender, race/ethnicity, asthma medication use, respiratory allergy history, atopic status, BMI, hour and day (of the week) of test, season, and temperature. Adjustment for short-term effects of air pollution using appropriate lag structure at each study period together with potential confounders and effect modifiers taken into consideration, will help assess the independent effects of long-term air pollution. Time independent covariates (∆Z) such as race/ethnicity effects will be assessed together with time-elapsed between the two child-specific yearly test dates (∆Age), while time-varying covariates (∆W) effects will be assessed considering all possible transitions or changes over time for categorical and continuous covariates respectively. The most appropriate linear distributed lag models will be chosen from all possible types of lag-based models for short-term effects of air pollution, and the model selection will be based on the Akaike Information Criterion (AIC). The confounding effect of ambient temperature will be tested using the lag structure selected for the short-term air pollutant effects. Potential effect modifiers such as gender, asthma status, respiratory allergy, baseline outcome status (obstruction status, FeNO levels) and season will be tested in the final model.

*Analytical method*: Two strategies will be employed in these analyses. The first is a cohort analysis starting with a disease-free cohort to explore new-onset of asthma, while the second will be a change-analysis to investigate the changes in the respiratory outcomes over time. To explore the association between the asthma score and pollutant concentrations both defined at follow-up, in a subpopulation reporting neither symptoms nor asthma at baseline, a negative binomial regression model (with a log link) will be used to account for extra-Poisson variation due to the distribution of the asthma score, with a majority of zeros. The result will be expressed as ratios of the mean asthma scores (RMS). The pollutants effect will be scaled for an increase of 10 μg/m^3^ higher concentration. The subpopulation of interest will be considered a sample being in all likelihood free of asthma at baseline. Thus, new onset of symptoms (a reflection of asthma incidence) might be interpreted following the occurrence of symptoms at follow-up. To account for the participant reporting only one of the symptoms at follow-up, further analysis will be performed by comparing those with none or only one symptoms (coded as participants free of symptoms) at follow-up with those with at least two symptoms. This will also be followed-up by considering those reporting none, one or two symptoms as participants free of symptoms, comparing them with participants reporting at least three symptoms.

Multiple linear regression will be used to assess the relationship between changes in levels of FeNO (∆FeNO) or percentage predicted FEV_1_ (∆%FEV_1_) and FEV_1_/FVC ratio (∆FEV_1_/FVC) between the two study periods and the corresponding changes in ‘long term’ and ‘short term’ air pollution and airborne pollen levels. A 12-month period prior to the day of tests will be used to assess the effects of changes in long term pollution levels, while adjusting for short term levels based on lags of up to 60 days prior to the day of tests. The model will assume a general form provided below;

$$ \Delta \mathrm{FeNO}\ \left(\Delta \%{\mathrm{FEV}}_1\ \mathrm{or}\ \Delta {\mathrm{FEV}}_1/\mathrm{FVC}\right)={\upbeta}_0\Delta \mathrm{Age}+{\upbeta}_1{\Delta \mathrm{AP}}^{\mathrm{LT}}+{\upbeta}_2{\Delta \mathrm{AP}}^{\mathrm{ST}}+{\upbeta}_3{\Delta \mathrm{P}}^{\mathrm{LT}}+{\upbeta}_4{\Delta \mathrm{P}}^{\mathrm{ST}}+{\upbeta}_5{\Delta \mathrm{Temp}}^{\mathrm{ST}}+{\upgamma}^{\mathrm{T}}\mathrm{Z}\ \mathrm{x}\ \Delta \mathrm{Age}+{\upgamma}^{\mathrm{T}}\Delta \mathrm{W}+\upvarepsilon $$

where ∆Age, ∆AP^LT^, ∆AP^ST^, ∆P^LT^, ∆P^ST^, and ∆Temp^ST^ denotes the time elapsed between the two tests, changes in long-term air pollution levels, short-term pollution, changes in long-term pollen levels, short-term pollen levels, and temperature levels, respectively. The change-on-change model will allow for exploring the determinants of change in FeNO or %FEV_1_ or FEV_1_/FVC rather than determinants of level of the actual FeNO or %FEV_1_ or FEV_1_/FVC. In an attempt to explore the change in obstructive pattern defined by reduced FEV_1_ less than 80% of the predicted value and a reduced FEV_1_/FVC ratio less than 0.8, a binary variable will be created with participant changing from non-obstructive airways to obstructive airways coded as 1; those without change in obstructive airways pattern also coded as 1; those with changes from obstructive airways to non-obstructive airways coded as 0; and those without change in non-obstructive airways pattern also coded as 0. Thus, a multiple logistic regression will be used to model the association between change in obstructive patterns and change in exposure taking into consideration confounding and effect modifiers.

### Analysis protocol for objectives 4

*Predictor*: Baseline FeNO levels.

*Outcome*: Crude incidence rates for new-onset asthma (defined by increasing ASS or presence of new asthma symptom) will be calculated by dividing the number of cases by the total person-years at risk. Follow-up is considered complete at the time of reported diagnosis for children who developed new-onset asthma.

*Covariates*: The potential confounders include; age, gender, race/ethnicity, asthma medication use, respiratory allergy history, atopic status, BMI, season, temperature including average ambient air pollution and airborne pollen levels on the day of the FeNO measurement.

*Analytical approach*: To investigate the relationship between baseline FeNO with new-onset asthma, incident rates will be calculated while exploring series of multivariate modelling approaches to adjust for potential confounders and heterogeneity of effects within subgroups of children. A cohort analyses starting with a disease-free cohort will be fitted to investigate the association between FeNO and new-onset asthma adjusting for possible confounders including lifetime history of wheezing. Fitted models with appropriate interaction terms with statistical significance tested by partial likelihood ratio test will be used to assess heterogeneity of associations among subgroups. Thereafter, stratified analyses will be performed in the presence of such significant interaction. Splines and piecewise cubic polynomials joined smoothly at a number of breakpoints (knots) will be used to explore the nonlinearity nature of FeNO effects.

## Discussion

This research intends to explore state of the art approaches in the characterization of exposure to ambient air pollutant to investigate its effects on children’s respiratory health (with a specific focus on asthma) in South African informal settlement areas, a heavily under researched setting. The research employs the application of more novel outcomes and exposure measures such as biological markers of airway inflammation (such as FeNO) in addition to traditional measures (spirometry, peak expiratory flow, asthma symptom score, and allergic sensitization to common inhalants) as well as ambient air pollution estimates derived from LUR modelling and GIS-based indicators in addition to dispersion modelling and air quality monitoring stations data. Additionally, the study measures pollen levels (mould spores, tree, grass and weed pollen) in the study areas supplemented with meteorological factors (such as wind direction, wind speed, temperature, humidity, pressure and sunshine) that may impact on pollutant levels and pollen counts. This study attempts to investigate the synergistic effect of air pollutant and pollen exposures on both new-onset childhood asthma and asthma exacerbations at the population level. Furthermore, this study promises to provide additional data for the Western Cape Province of South Africa to further explore the association between ambient air pollution and respiratory morbidity (childhood asthma) in South Africa. The study will generate crucial data on air pollution and asthma in low income settings in sub-Sahara Africa that is lacking in the international literature.

A demonstrable positive association between ambient air pollution and asthma in children in the Western Cape would support the importance and urgency of improving ambient air quality in the Province and particularly in the study areas. This would contribute towards highlighting the need for ongoing ambient air quality monitoring and strategies for the continued reduction of air pollutants from likely sources such as traffic, passive smoking, industries, and domestic fuels. There is potential to use these information towards increasing and promoting asthma awareness and asthma education in communities with a high prevalence of asthma including the study communities. It is believed that such public health promotion initiatives would assist in reducing associated asthma morbidity. Education programmes will emphasize the need for household environmental management including indoor dust control, ownership of certain pets, pest and bio-aerosol control, prevention of smoking indoors and actively discouraging adults from smoking particularly around children or in the vicinity of their rest, recreational or study areas. These measures will, in conjunction with ambient air quality management, result in an improved quality of life of asthmatics, particularly those with persistent asthma including those who are susceptible. Since air pollutants have been shown to influence the allergenic content in plants by influencing pollen production and allergenic protein in pollen grains, abatement of air pollution will mitigate effects of climate change, allergen exposures, air quality and health status of vulnerable populations.

Furthermore, the findings from this project will also help the Department of Environmental Affairs and Development Planning (DEA&DP) in the Western Cape, South Africa to develop regional and spatial plans that consider these findings (with regard to location of industries and major roads close to residential areas), and review environmental exposure standards. This will contribute towards highlighting the importance of ongoing monitoring of air pollution pollen exposures near urban areas and near schools, with results communicated to the public in a concise and understandable means. This would contribute towards the protection of public health during periods of high levels of ambient air pollution whereby authorities can use this information as part of their decision-making processes.

## Abbreviations

AIC: Akaike information criterion
ATS: American thoracic society
BMI: Body mass index
CAMx: Comprehensive air quality model
DALY: Disability-adjusted life years
DAS: Data acquisition system
DEA&DP: Department of Environmental Affairs and Development Planning
ERS: European respiratory society
FeNO: Fractional-exhaled nitric oxide
FEV_1_: Forced-expiratory volume in one second
FVC: Forced vital capacity
GAM: Generalized additive models
GEE: Generalized estimating equation
IgE: Immunoglobulin E
IQR: Interquartile range
ISAAC: International study of asthma and allergy in childhood
LMI: Low and middle income countries
LUR: Land-use regression
NIOH: National institute of occupational health
NO_2_: Nitrogen dioxide
O_3_: Ozone
OR: Odds ratio
PEF: Peak expiratory flow
PM_1.0_: Particulate matter with diameter of 1.0 microns
PM_10_: Particulate matter with diameter of 10 microns
PM_2.5_: Particulate matter with diameter of 2.5 microns
RD: Risk difference
RMS: Ratio of mean score
SAWS: South Africa Weather Services
SO_2_: Sulphur dioxide
UCT: University of Cape Town
